# Effects of different electrical stimulation on depression: a protocol for systematic review and network meta-analysis

**DOI:** 10.3389/fpsyt.2025.1684994

**Published:** 2025-11-21

**Authors:** Shaoyan Yao, Song Li, Qinying Ran, Tao Zhu, Raoting Zhang, Lei Zhou, Jianmei Zhang

**Affiliations:** 1 Mental Hospital of Yunnan Province, Kunming, China; 2 Affiliated Mental Health Center of Kunming Medical University, Kunming, China; 3 Yunnan University of Chinese Medicine, Kunming, China; 4 The Second Clinical Medical College, Heilongjiang University of Chinese Medicine, Harbin, China; 5 Yinjiang Autonomous County Traditional Chinese Medicine Hospital, Tongren, China; 6 Guizhou University of Traditional Chinese Medicine, Guiyang, China; 7 Qujing Traditional Chinese Medicine Hospital, Qujing, China; 8 Yan’an Hospital Affiliated to Kunming Medical University, Kunming, China

**Keywords:** depression, electrical stimulation, network meta-analysis, randomized control trial, protocol

## Abstract

**Objective:**

Depression is a common mental disorder characterized by persistent depressive mood, anhedonia, and diminished interest, severely impacting patients’ quality of life and social functioning. Traditional treatments exhibit significant interindividual variability in efficacy, adverse reactions, uncertain long-term outcomes, high relapse rates upon discontinuation, prolonged treatment cycles, and substantial economic burdens. Electrical stimulation therapy represents a potential intervention, yet its efficacy and safety remain inconsistent across studies. This study aims to analyze the efficacy and safety of different electrical stimulation modalities in treating patients with depression, quantitatively assessing their comparative advantages and potential clinical benefits.

**Methods:**

We will comprehensively search 13 Chinese and English databases including PubMed, Embase, Cochrane Library, Web of Science, Scopus, ClinicalTrials.gov, WHO-ICTRP, OpenGrey, ProQuest China National Knowledge Infrastructure, VIP Database, Wanfang Database, and China Biomedical Database. The search time will be from the establishment of each database to August 1, 2025; 2 independent reviewers will use Cochrane the Risk of Bias Assessment Tool (Second Edition) to evaluate the methodological quality and potential bias of included studies; the primary outcome indicators will be the overall effectiveness rate, Hamilton Depression Rating Scale (HDRS) score, and Self-Rating Depression Scale (SDS) score. The secondary outcome indicators are the cure rate, Patient Health Questionnaire-9 (PHQ-9) score, adverse event rate, Side Effect Rating Scale (SERS) score, and Side Effects Scale (TESS) score; STATA will be used. Software data synthesis will be performed using a random effects model for network meta-analysis to compare the effectiveness and safety of different electrical stimulation therapies. The surface under the cumulative ranking curve is also used to indicate the likelihood of benefits and harms of an intervention. The strength of the evidence will be assessed by grading the Recommendations, Assessment, Development, and Evaluation framework.

**Justification:**

This protocol is designed to provide evidence supporting the efficacy of electromagnetic stimulation therapy in alleviating clinical symptoms in patients with depression. Furthermore, these findings will yield a theoretical foundation and actionable reference for clinicians to optimize therapeutic strategies for depression. Depression, Electrical stimulation, network meta-analysis, randomized control trial, protocol.

**Trial registration:**

PROSPERO https://www.crd.york.ac.uk/prospero/ registration number: CRD420251103264.

## 1 Introduction

Depression, a prevalent mental health disorder characterized by persistent low mood, anhedonia, and fatigue, has emerged as a major global public health concern (1, 2). The World Health Organization (WHO) epidemiological data reveal that over 350 million people worldwide are affected by depression, with its prevalence showing a consistent upward trend. Currently, the global prevalence of depression is 3.2% (3). As the second leading contributor to the global disease burden, depression is primarily characterized by persistent and significant mood disturbances. Projections suggest that by 2030, depression will become the leading contributor to the global disease burden and the primary cause of non-fatal health loss (4). This condition imposes substantial socioeconomic burdens, and its association with suicidal ideation is of particular concern: more than 10% of patients report suicidal thoughts, while a subset exhibit explicit suicidal intent (5).

Contemporary management of depression primarily involves non-pharmacological interventions—including biopsychosocial approaches, acupuncture, and music therapy—alongside pharmacological treatments such as selective serotonin reuptake inhibitors (SSRIs), serotonin-norepinephrine reuptake inhibitors (SNRIs), serotonin modulators, tricyclic antidepressants (TCAs), and monoamine oxidase inhibitors (MAOIs) (2, 6). Although these treatments have demonstrated efficacy in alleviating depressive symptoms, their mechanisms of action—primarily inhibiting neurotransmitter reuptake and subsequent increases in synaptic neurotransmitter concentrations—have notable limitations. These limitations include adverse reactions of varying severity, development of drug resistance, and high rates of symptom recurrence (2, 7, 8). Consequently, there is an urgent need to identify complementary treatment strategies for patients with depression that exhibit enhanced efficacy, improved safety, and fewer side effects.

Current evidence indicates that electrical stimulation methods can alleviate depression by modulating the function of key neural networks—including the Default Mode Network (DMN), Sensorimotor Network (SMN), Cognitive Control Network (CCN), and Visual Network (VN)—as well as the limbic system (9–12). In addition to directly regulating neural network function, the therapeutic effects of electrical stimulation also involve targeting multiple biological system dysregulations linked to depression, as detailed below: First, monoamine imbalance (e.g., serotonin [5-HT]) in the nervous system is a common pathological feature of depression. Reduced 5-HT neurotransmission is associated with core emotional symptoms, such as persistent sadness and hopelessness (13). Electrical stimulation can alleviate patients’ clinical symptoms by regulating 5-HT levels (14, 15). Second, elevated pro-inflammatory cytokines disrupt neuroplasticity and interfere with monoamine metabolism, thereby exacerbating fatigue, psychomotor retardation, and mood disturbances (16, 17). Recent studies have confirmed that electrical stimulation can inhibit the production of interleukin-6 (IL-6) and tumor necrosis factor-α (TNF-α) by activating the cholinergic anti-inflammatory pathway, thereby facilitating recovery from inflammation-related depression (18). Furthermore, chronic hyperactivity of the hypothalamic- pituitary- adrenal (HPA) axis impairs hippocampal neurogenesis and exacerbates emotional dysregulation, including heightened suicidal ideation (19). Electrical stimulation can regulate HPA axis function by reducing cortisol secretion, thereby improving emotional function (20). Finally, neurotrophic factors are critical for maintaining neuronal survival and synaptic plasticity. Downregulation of brain-derived neurotrophic factor (BDNF) in the hippocampus and prefrontal cortex is strongly associated with cognitive impairments in depression (21). Electrical stimulation can upregulate BDNF expression, thereby improving cognitive function in patients with depression (22).

Current electrical stimulation techniques include electroacupuncture (9), transcutaneous acupoint electrical stimulation (11), brain electrical bionic stimulation (23), dense cranial electroacupuncture stimulation (24), and transcutaneous auricular vagus nerve electrical stimulation (25). Research has confirmed that electrical stimulation therapy for depression not only exerts adjunctive therapeutic efficacy in alleviating depressive symptoms but also offers potential clinical benefits, including mitigating medication-related adverse reactions and enhancing patient acceptance and tolerance of treatment (26–28). However, a comprehensive systematic network meta-analysis (NMA) assessing the comparative clinical efficacy of different electrical stimulation therapies for depression is currently lacking in the existing literature.

In medical research, conventional meta-analysis methods are limited to direct comparisons between two interventions, thereby restricting their analytical scope. The development of network meta-analysis (NMA) —a sophisticated statistical technique— has addressed this limitation by synthesizing both direct and indirect comparative evidence, thereby enabling comprehensive evaluation of multiple therapeutic interventions. This approach maximizes the use of existing research data, providing researchers and clinicians with more robust, reliable evidence to inform clinical decision-making and establishing a scientific basis for identifying optimal treatment strategies for patients (29). Leveraging this methodological advancement, the present study will use NMA to systematically compare the efficacy and safety profiles of various electrical stimulation therapies for treating depression. By quantitatively assessing their relative advantages and potential benefits, this study aims to generate valuable clinical insights to guide therapeutic decision-making for both clinicians and patients.

## 2 Methods

### 2.1 Objectives

#### 2.2.1 Efficacy evaluation

This study will employ systematic review and network meta-analysis methodologies to quantitatively assess the therapeutic efficacy of various electrical stimulation therapies for depression. It will aim to elucidate the advantages and limitations of different intervention strategies in alleviating depressive symptoms, thereby providing robust scientific evidence to inform clinical decision-making.

#### 2.2.2 Safety evaluation

This study will systematically collate and analyze the incidence rates of adverse events associated with different electrical stimulation modalities for depression. It will assess the safety of these interventions, providing clinicians and patients with reliable safety information.

### 2.2 Study registration

This systematic review protocol was meticulously developed in full compliance with the Preferred Reporting Items for Systematic Reviews and Meta-Analyses (PRISMA-P) guidelines (30) and the PRISMA extension for network meta-analyses (31). To ensure methodological transparency and reproducibility, the protocol was prospectively registered in the International Prospective Register of Systematic Reviews (PROSPERO; registration number: CRD420251103264). The completed PRISMA-P checklist is included as **Supplementary Material** in the **Appendix S1 File**.

### 2.3 Data source and search strategy

The search strategy for this investigation will be meticulously developed to ensure comprehensive coverage of pertinent literature. A dual approach incorporating both Medical Subject Headings (MeSH) and free-text search terms will be employed to optimize the precision and inclusivity of the search results. The Boolean logic-based search protocol will be designed to encompass all available literature from the inception of each database through August 1, 2025. The search will be conducted across multiple databases, including PubMed, Embase, the Cochrane Library, Web of Science, Scopus, ClinicalTrials.gov, WHO International Clinical Trials Registry Platform(WHO-ICTRP), OpenGrey, ProQuest, China National Knowledge Infrastructure (CNKI), VIP Database, Wanfang Database, and the Chinese Biomedical Database (CBM). The specific search strategies for both Chinese and English databases will be delineated in the **Appendix S2 File**.

### 2.4 Eligibility criteria

#### 2.4.1 Study design

This systematic review will exclusively incorporate peer-reviewed journal articles reporting randomized controlled trials (RCTs), with no restrictions imposed on language or geographic origin. Studies employing non-randomized designs, including case-control studies, cohort studies, case reports, and other observational or non-experimental methodologies, will be excluded from the analysis.

#### 2.4.2 Participant characteristics

Participants meeting the inclusion criteria must be definitively diagnosed with depression by a qualified psychiatrist in accordance with the diagnostic criteria for depression outlined in either the International Classification of Diseases, 11th Revision (ICD-11) or the Diagnostic and Statistical Manual of Mental Disorders, Fifth Edition Text Revision (DSM-5-TR) (32). Our study will impose no age restrictions on participants and will exclude individuals with bipolar disorder, primary anxiety disorders, or those presenting with comorbid conditions.

#### 2.4.3 Intervention protocols

The intervention group will undergo fourteen types of electrical stimulation therapy, categorized according to predefined classifications as follows: Central Nervous System (CNS) Targeting Methods: electroconvulsive therapy, repetitive transcranial magnetic stimulation, transcranial direct current stimulation, transcranial pulsed electrical stimulation, transcranial alternating current stimulation, electroencephalographic bionic stimulation, and transcranial neural electrical stimulation (all directly acting upon the cerebral cortex or CNS pathways); Peripheral Nerve Targeting Modalities: vagus nerve stimulation, transcutaneous vagus nerve stimulation, and vestibular nerve stimulation (acting on peripheral nerves to modulate neural circuits); Acupoint/Soft Tissue Targeting Modalities: electroacupuncture, auricular electroacupuncture, and transcutaneous acupoint electrical stimulation (combining traditional acupoint theory with electrical stimulation). Neuromuscular-targeted modalities: Neuromuscular electrical stimulation (focusing on neuromuscular junctions to enhance motor function). Electrical stimulation treatment courses must be maintained for at least two weeks, with no restrictions on follow-up duration.

#### 2.4.4 Control group protocols

In accordance with the network connectivity requirements of network meta-analyses, the control groups were categorized into a three-tier hierarchical framework based on intervention activity and clinical routine to reduce heterogeneity and ensure analytical validity: Tier 1: Placebo/sham intervention group, including pharmacologically inactive placebo medications and sham biophysical therapies; Level 2: Treatment-as-usual group, employing standard treatment protocols as specified in the Primary Care Guidelines for Major Depressive Disorder (33), including pharmacotherapy, psychotherapy, biophysical therapies, phototherapy, exercise therapy, and reading therapy; Tier 3: Active intervention group, referring to interventions beyond conventional treatment with demonstrated efficacy (e.g., novel targeted medications, intensive psychotherapy), included solely to maintain network connectivity where direct comparative evidence exists.

#### 2.4.5 Types of outcomes

##### 2.4.5.1 Primary outcomes

The primary outcomes of this study will be defined as the overall response rate to electrical stimulation therapy for depression, assessed using the Hamilton Depression Rating Scale (HDRS) (34). The overall response rate will be calculated as the proportion of participants who will exhibit a positive treatment response relative to the total number of enrolled subjects (Overall Response Rate = Number of Responders/Total Number of Subjects). The HDRS, a widely validated instrument, will quantify the severity of depressive symptoms, particularly in evaluating the efficacy of pharmacological and psychotherapeutic interventions. The 17-item version (HAMD-17), with a scoring range of 0–50, will be utilized, where higher scores indicate greater symptom severity. While the HDRS demonstrates high internal consistency and inter-rater reliability, certain items exhibit limited test-retest reliability. Additionally, this study will establish primary outcome measures comprising response rate and remission rate, both defined using standardized HDRS scoring criteria: response rate is defined as a ≥50% reduction in the subject’s HDRS score from baseline at treatment completion; remission rate is defined as a total HDRS score ≤7 at treatment completion, consistent with the established criterion for symptom remission in clinical depression research.

##### 2.4.5.2 Secondary outcomes

Secondary outcome measures will encompass the Patient Health Questionnaire-9 (PHQ-9), quality of life, and safety parameters. Additional assessment criteria include the Side Effects Rating Scale (SERS), the Treatment-Emergent Symptoms Scale (TESS), the number of subjects withdrawing from the study for any reason, and the response rate.

### 2.5 Study selection

**Figure 1:**
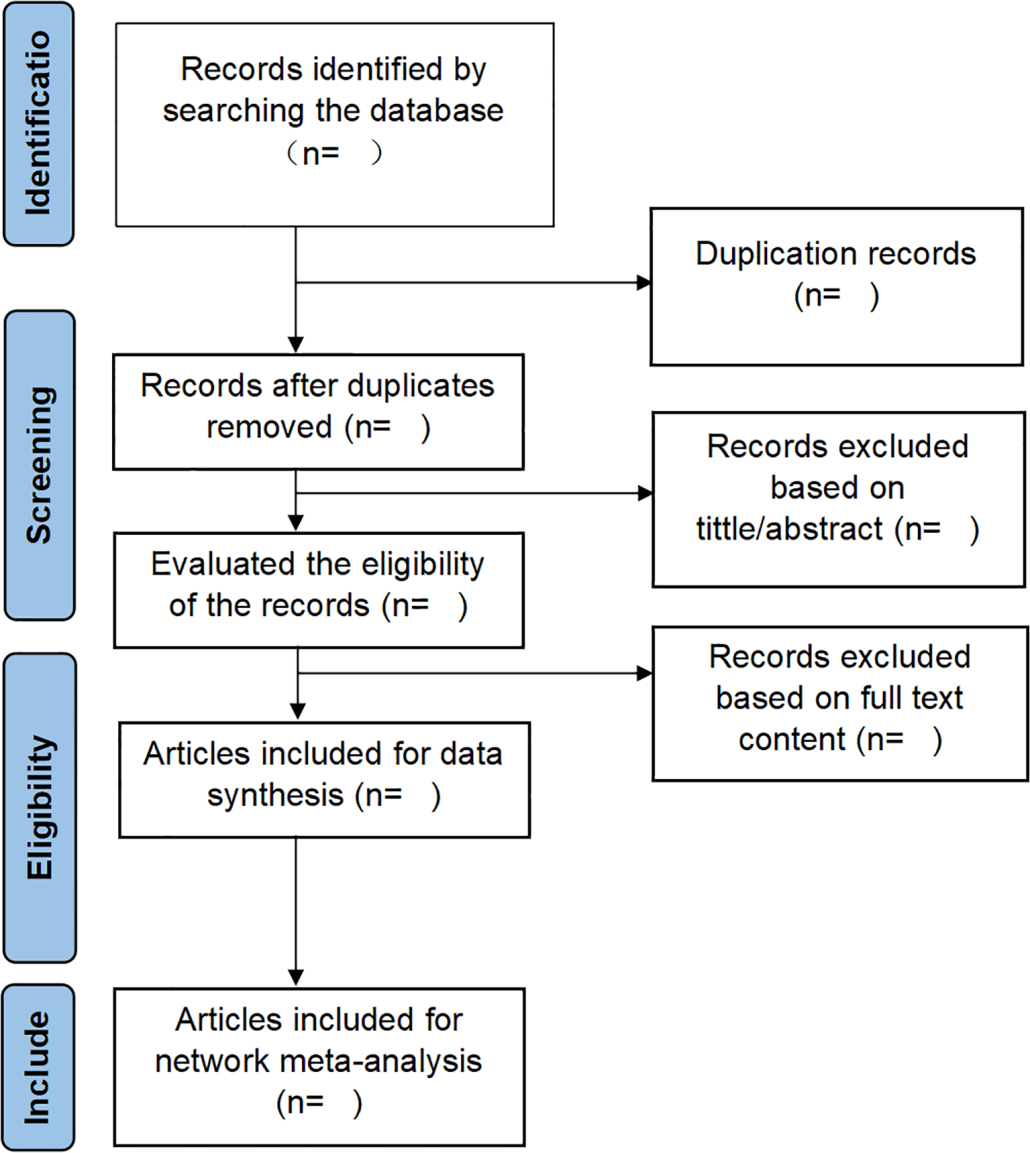
The details of the selection process.

In the present investigation, EndNote X9 software will screen search results systematically. Two independent reviewers will conduct the screening process in duplicate, followed by cross-verifying outcomes. The screening procedure will be rigorously executed in accordance with predefined inclusion and exclusion criteria, with relevant studies identified through a comprehensive evaluation of titles, abstracts, and full-text articles. In duplicate publications, the original source will be prioritized for reference. For studies presenting incomplete data or ambiguous details, direct communication will be initiated with the first or corresponding authors to obtain necessary clarifications. Any discrepancies arising between the two reviewers during the screening process will be resolved through consultation with a third reviewer, with final decisions reached through consensus-based discussion. The PRISMA flowchart (**Figure 1**) will serve as a methodological framework, providing a transparent and systematic representation of the screening process.

### 2.6 Data extraction

For this investigation, two independent reviewers will systematically perform data extraction using a standardized Excel-based data collection template to retrieve pertinent information from studies included in the final analysis. A clear hierarchy of data extraction will be established, prioritizing the extraction of core study information (e.g., intervention measures, outcome indicators) followed by the gradual extraction of detailed information (e.g., sample size stratification, treatment parameters). In instances where data will be incomplete or ambiguous, corresponding authors will be contacted via electronic correspondence to acquire missing information or clarify ambiguities. Before analytical procedures, all extracted data will undergo rigorous cross-verification, and any identified discrepancies will be resolved through consensus discussions among the review team to ensure data integrity and consistency. The extracted dataset will encompass the following domains (1): Study characteristics, including authorship, publication year, geographic location, study design, and research institution (2); Participant demographics and clinical profiles, including sample size, gender distribution, age range, diagnostic criteria, disease characteristics (including disease duration, depression type, etc.), pharmacological interventions, treatment protocols, and follow-up procedures (3); Intervention and comparator specifications, detailing electrical stimulation modalities, treatment duration, frequency, session parameters, and primary stimulation characteristics (4); Outcome measures, incorporating all quantitative assessments, adverse event documentation, and temporal measurement points. The study will include all continuous data in calculations of pre- and post-treatment value changes, including determining differences between post-treatment and pre-treatment metrics. If these calculations are not presented in the original text, they will be computed follows, with corr typically set to 0.5.


$$\begin{array}{c} SD_{E}change=\\ \sqrt{SD_{E\;baseline}^{2}+SD_{E\;final}^{2}-(2\times Corr\times SD_{E}\;baseline\times SD_{E}\;final)} \end{array}$$

$${\text{Mean}}_{E\;change}={\text{Mean}}_{E\;final}-{\text{Mean}}_{E\;baseline}$$

If the evaluation results in the literature are inconsistent, we will perform data conversion and standardization. When data extraction is disputed, we invite a third party to participate in discussions to resolve any issues. We will contact the corresponding author or co-authors to obtain raw data when necessary. Standardized methods for imputing missing statistical information will be adopted: linear interpolation will be used for continuous outcomes, and proportional approximation will be used for dichotomous outcomes. For the outcome measurement timepoints, we will prespecify primary and secondary timepoints consistent with clinical practice for electroconvulsive therapy for depression: the primary timepoint for efficacy outcomes, with secondary timepoints used for subgroup analyses to explore long-term effects. For studies reporting multiple time points for the same outcome, we will prioritize data from the pre-specified primary time point for the main analysis. If primary time point data is missing, we will estimate values using linear interpolation (for continuous outcomes) or proportional approximation (for dichotomous outcomes). All preprocessing decisions (e.g., data conversion, missing value imputation, timepoint prioritization) will be clearly documented in the appendix of the study protocol.

### 2.7 Safety outcomes

Safety outcomes primarily refer to adverse events (AEs), including symptoms such as palpitations, nausea, and vomiting, which will be assessed using a ternary scoring system (0 = absent, 1 = present). Where necessary, we will conduct a pooled analysis of AEs frequency categorized by severity: mild (minimal discomfort requiring no treatment), moderate (discomfort requiring over-the-counter medication), and severe (requiring urgent care or discontinuation of therapy).

### 2.8 Risk of bias assessment

The risk of bias assessment in this investigation will be independently performed by two reviewers utilizing the Cochrane Risk of Bias tool (RoB 2) (35), a validated instrument designed for randomized controlled trials. To mitigate the potential impact of subjective factors on the assessment outcomes, reviewers will undergo comprehensive training and be provided with detailed operational guidelines before the formal initiation of the study. The RoB 2 tool encompasses five critical domains for evaluation: bias arising from the randomization process, bias due to deviations from intended interventions, bias due to missing outcome data, bias in outcome measurement, and bias in the selection of reported results. Reviewers will categorize each predefined outcome included in the study as “low”, “some concern”, or “high” risk of bias, thereby clearly reflecting the methodological reliability of the research. Reviewer discrepancies will be systematically discussed and resolved through consensus within the review panel. Finally, the bias risk assessment results will be graphically summarized using a bias risk map to facilitate visual interpretation.

### 2.9 Data synthesis

#### 2.9.1 Pairwise meta-analysis

In the present investigation, we will evaluate clinical heterogeneity and its magnitude across the included studies by systematically analyzing variations in participant demographics, baseline characteristics, intervention protocols, and outcome measures, employing the I² statistic alongside p-values for quantification. The interpretation of I² values will adhere to established thresholds: I² values exceeding 25%, 50%, and 75% will be classified as indicative of low, moderate, and high statistical heterogeneity, respectively. In instances where the I² value surpasses 50%, signifying substantial heterogeneity, a random-effects model will be implemented for the meta-analysis. Conversely, when the I² value remains at or below 50%, a fixed-effects model will be utilized to estimate the pooled effect size (36).

#### 2.9.2 Network meta-analysis

We will perform statistical analyses using Stata 18.0 software and select a random-effects model based on a frequency framework to analyze direct and indirect evidence for all interventions. Specifically, indirect comparisons will be based on the transmission hypothesis: inferring the unknown difference between A and B by leveraging known differences between A and C, and B and C, where C represents a third intervention. Concurrently, to validate the robustness of the transmission hypothesis and explore sources of heterogeneity, assessments will be conducted via stratified analysis or meta-regression of effect modifiers. The specific modifiers included are: 1. Patient baseline characteristics: baseline disease severity, disease duration, age; 2. Intervention implementation parameters: electrical stimulation- related operational parameters (frequency, stimulation duration, stimulation intensity). By comparing effect size differences within distinct modifier subgroups, we will determine whether these factors exert a significant influence on intervention effects. This will provide support for the validity of the transmission hypothesis and elucidate potential sources of outcome heterogeneity. Where closed loops exist, we shall employ node partitioning to report and examine inconsistencies. For continuous data, we will calculate pooled standardized mean differences (SMD) across assessment scales and estimate mean differences (MD) for each outcome measure using unit consistency as the criterion. We will estimate pooled risk ratios (RR) and calculate corresponding 95% confidence intervals (95% CI) for categorical data. First, we will use the “networkplot” command to generate a network relationship diagram illustrating quantitative relationships between studies and interventions. In the network diagram, individual nodes represent distinct interventions, while connecting edges denote pairwise comparisons between interventions. Node sizes will be scaled proportionally to corresponding sample sizes, with larger nodes indicating interventions involving larger sample sizes. We will assess consistency between “direct comparison evidence” and “indirect comparison evidence” using the “mvmeta inconsistency” global inconsistency test. If the network diagram forms a closed loop, we will employ the node splitting method via the “network sidesplit all, tau” command to test for inconsistency. Second, we will synthesize data using a random-effects model of network meta-analysis to compare the relative efficacy of different electrical stimulation therapies. Finally, the “sucra prob*” command will rank the effects of different interventions and generate cumulative probability plots. The surface under the cumulative ranking curve (SUCRA) will represent interventions’ relative advantages and disadvantages. A higher SUCRA score indicates greater intervention effectiveness. Finally, the “netfunnel” command will generate funnel plots to assess publication bias and small-sample evaluation for included studies. Findings will be presented visually.

#### 2.9.3 Subgroup and sensitivity analyses

This study will be designed to systematically investigate and mitigate potential sources of heterogeneity and inconsistency in network meta-analyses through comprehensive subgroup and sensitivity analyses. Subgroup analyses will be stratified according to predefined factors, including intervention type (e.g., distinct modalities of electrical stimulation therapies) and participant type (e.g., postpartum depression, post-stroke depression, adolescent depression, geriatric depression). Examining intervention types will elucidate various electrical stimulation therapies’ differential efficacy and safety profiles. In contrast, the analysis of participant types will evaluate the influence of population-specific characteristics on intervention outcomes.

Sensitivity analysis will assess the robustness of study results by evaluating the impact of study quality and other potential sources of heterogeneity. We will conduct a study-by-study exclusion analysis: after removing one study at a time, we will refit the model and calculate the magnitude of change in the SUCRA (Cumulative Ranked Probability) values for each intervention. The results will be considered robust if all changes are <15%. Studies with lower methodological quality will be excluded to determine their impact on the overall results. Furthermore, suppose the preliminary analysis reveals significant heterogeneity (I²> 50%). In that case, meta-regression analysis will explore the association between study-level covariates—such as different electrical stimulation frequencies and effect sizes—to address heterogeneity and inconsistency. By incorporating covariates into the meta-regression model, we will identify and quantify the contribution of potential sources of heterogeneity, thereby providing a more nuanced understanding of the factors contributing to the variability observed in the network meta-analysis results.

### 2.10 Publication bias

In the present investigation, publication bias will be systematically evaluated through funnel plot analysis and Egger’s regression test when the number of included studies reaches a minimum threshold of 10. The funnel plot methodology will offer a graphical assessment of potential publication bias by plotting effect sizes against their corresponding standard errors, with asymmetry in the distribution of studies indicating possible bias. Complementing this visual approach, Egger’s linear regression test will quantitatively assess publication bias by examining whether the regression intercept significantly deviates from zero through a weighted regression analysis. It should be noted that the statistical power of Egger’s test may be limited when applied to smaller study samples (n<10); however, the combined application of both funnel plot analysis and Egger’s test will provide a robust and comprehensive approach to publication bias assessment in meta-analytic studies (37, 38).

### 2.11 Grading the strength of evidence

In this investigation, we plan to employ the Grading of Recommendations, Assessment, Development, and Evaluation (GRADE) framework, utilizing the online GRADE tool (https://gradepro.org/) to provide methodological support for systematically and separately assessing the credibility of each outcome across three evidence types in NMA: direct evidence, indirect evidence, and network evidence. Definitions of the three evidence types are clarified first: direct evidence refers to effect estimates from head-to-head randomized controlled trials (RCTs) comparing two interventions; indirect evidence denotes effect estimates inferred via a common comparator; network evidence represents integrated effect estimates synthesizing direct and indirect evidence for all interventions in the NMA network. Strict adherence to the grading criteria may result in downgrading of the quality rating due to universal factors (risk of bias, imprecision, publication bias, applicable to all three evidence types) and type-specific factors (inconsistency of effect sizes for direct evidence, transitivity plausibility for indirect evidence, network consistency via node-splitting and structural completeness for network evidence). Evidence strength will be categorized into four distinct levels: high, moderate, low, and very low. High-quality evidence will indicate a high likelihood that the true effect is close to the estimated effect, warranting strong recommendations for clinical practice. Conversely, very low-quality evidence will reflect substantial uncertainty about the true impact, suggesting potential significant differences from the estimated effect (39).

### 2.12 Ethics and dissemination

This investigation will exclusively utilize data obtained from publicly accessible databases, precluding direct human subjects or public participation involvement. Since that all data will be derived from previously published literature, this study will be exempt from requiring formal ethical approval from an institutional review board. The research outcomes will undergo rigorous peer review before submission for publication in a reputable scientific journal and will be disseminated through presentations at international academic conferences.

## 3 Discussion

Depression, a prevalent mood disorder, manifests as persistent low mood accompanied by symptoms including sadness, irritability, sleep disturbances, appetite loss, constipation, and diminished self-esteem, with severe cases potentially exhibiting self-harm or suicidal behaviors (40, 41). While conventional therapeutic approaches, including pharmacotherapy and psychotherapy, remain cornerstone treatments, their limitations—such as adverse effects, non-response in specific patient populations, and protracted treatment durations—necessitate the exploration of alternative interventions. Electrical stimulation therapies, including electroacupuncture and electroconvulsive therapy, have demonstrated efficacy in alleviating depressive symptoms, with multiple paired meta-analyses indicating superior outcomes compared to non-electrical stimulation interventions. This study employs systematic reviews and network meta-analysis (NMA) to establish a comprehensive framework for evaluating the efficacy and safety of diverse electrical stimulation modalities in depression treatment. By synthesizing extant evidence, the study aims to furnish evidence-based guidance for clinical decision-making, facilitate the selection of optimal treatment strategies for patients, and inform future research directions in electrical stimulation therapy for depression.

Several limitations warrant consideration. First, including heterogeneous electrical stimulation therapies with varying parameters (e.g., frequency, intensity, duration) may introduce inter-study heterogeneity, potentially compromising the robustness of the findings. Second, the study population encompasses diverse depressive subtypes (e.g., post-stroke depression, postpartum depression, adolescent depression, major depressive disorder) and comorbid conditions, which may influence treatment outcomes. Finally, restricting trials published in Chinese and English may introduce publication bias. To address these limitations and enhance the reliability and validity of the conclusions, subgroup and sensitivity analyses will be conducted to explore potential sources of heterogeneity. Additionally, the quality of evidence will be rigorously assessed using the GRADE (Grading of Recommendations, Assessment, Development, and Evaluations) criteria.

## References

[1] Hasin DS Sarvet AL Meyers JL Saha TD Ruan WJ Stohl M . Epidemiology of adult dsm-5 major depressive disorder and its specifiers in the United States. JAMA Psychiat. (2018) 75:336–46. doi: 10.1001/jamapsychiatry.2017.4602, PMID: 29450462 PMC5875313

[2] Malhi GS Bassett D Boyce P Bryant R Fitzgerald PB Fritz K . Royal Australian and New Zealand college of psychiatrists clinical practice guidelines for mood disorders. Aust N Z J Psychiatry. (2015) 49:1087–206. doi: 10.1177/0004867415617657, PMID: 26643054

[3] Kou Y Li Z Yang T Shen X Wang X Li H . Therapeutic potential of plant iridoids in depression: a review. Pharm Biol. (2022) 60:2167–81. doi: 10.1080/13880209.2022.2136206, PMID: 36300881 PMC9621214

[4] Varghese SP Florentin OD Koola MM . Role of spirituality in the management of major depression and stress-related disorders. Chronic Stress (Thousand Oaks). (2021) 5:227933408. doi: 10.1177/2470547020971232, PMID: 33506156 PMC7812390

[5] Nikolin S Owens K Francis-Taylor R Chaimani A Martin DM Bull M . Comparative efficacy, cognitive effects and acceptability of electroconvulsive therapies for the treatment of depression: protocol for a systematic review and network meta-analysis. BMJ Open. (2022) 12:e068313. doi: 10.1136/bmjopen-2022-068313, PMID: 36549738 PMC9772645

[6] Zhang J Zhao Y Li H Yang Y Tang Q . Effectiveness of acupuncture plus music therapy for post-stroke depression: systematic review and meta-analysis. Med (Baltimore). (2024) 103:e39681. doi: 10.1097/MD.0000000000039681, PMID: 39287303 PMC11404909

[7] Ruhe HG Horikx A van Avendonk M Groeneweg BF Woutersen-Koch H . Tapering of ssri treatment to mitigate withdrawal symptoms. Lancet Psychiat. (2019) 6:561–2. doi: 10.1016/S2215-0366(19)30182-8, PMID: 31230677

[8] Carreno FR Frazer A . Vagal nerve stimulation for treatment-resistant depression. Neurotherapeutics. (2017) 14:716–27. doi: 10.1007/s13311-017-0537-8, PMID: 28585221 PMC5509631

[9] Boontra Y Thanetnit C Phanasathit M . Effects of electroacupuncture on cognitive symptoms in major depressive disorder: a pilot study and randomized controlled trial. F1000Res. (2024) 13:479. doi: 10.12688/f1000research.146897.4, PMID: 39620153 PMC11605169

[10] Wang X Luo P Zhang L Sun J Cao J Lei Z . Altered functional brain activity in first-episode major depressive disorder treated with electro-acupuncture: a resting-state functional magnetic resonance imaging study. Heliyon. (2024) 10:e29613. doi: 10.1016/j.heliyon.2024.e29613, PMID: 38681626 PMC11053281

[11] Wang H BZ Y Guo Q Jing ZY . Effect of transcutaneous electrical acupoint stimulation combined with epidural labor analgesia on postpartum depression. Zhen Ci Yan Jiu. (2021) 46:231–4. doi: 10.13702/j.1000-0607.200249, PMID: 33798297

[12] Kapadia N Zivanovic V Moineau B Downar J Zariffa J Popovic MR . Functional electrical stimulation of the facial muscles to improve symptoms in individuals with major depressive disorder: pilot feasibility study. BioMed Eng Online. (2019) 18:109. doi: 10.1186/s12938-019-0730-6, PMID: 31727068 PMC6857333

[13] Mouri A Ikeda M Koseki T Iwata N Nabeshima T . The ubiquitination of serotonin transporter in lymphoblasts derived from fluvoxamine-resistant depression patients. Neurosci Lett. (2016) 617:22–6. doi: 10.1016/j.neulet.2016.01.064, PMID: 26845564

[14] Duan D Tu Y Yang X Liu P . Electroacupuncture restores 5-ht system deficit in chronic mild stress-induced depressed rats. Evid Based Complement Alternat Med. (2016) 2016:7950635. doi: 10.1155/2016/7950635, PMID: 27994633 PMC5141535

[15] Han X Wu H Yin P Chen Z Cao X Duan Y . Electroacupuncture restores hippocampal synaptic plasticity via modulation of 5-ht receptors in a rat model of depression. Brain Res Bull. (2018) 139:256–62. doi: 10.1016/j.brainresbull.2018.03.004, PMID: 29524471

[16] Kim YK KS N AM M Leonard BE . The role of pro-inflammatory cytokines in neuroinflammation, neurogenesis and the neuroendocrine system in major depression. Prog Neuropsychopharmacol Biol Psychiatry. (2016) 64:277–84. doi: 10.1016/j.pnpbp.2015.06.008, PMID: 26111720

[17] Iwata M KT O Duman RS . The inflammasome: pathways linking psychological stress, depression, and systemic illnesses. Brain Behav Immun. (2013) 31:105–14. doi: 10.1016/j.bbi.2012.12.008, PMID: 23261775 PMC4426992

[18] Yue N Li B Yang L QQ H HJ H YL W . Electro-acupuncture alleviates chronic unpredicta ble stress-induced depressive- and anxiety-like behavior and hippocampal neuroinflammation in rat model of depression. Front Mol Neurosci. (2018) 11:149. doi: 10.3389/fnmol.2018.00149, PMID: 29946236 PMC6007169

[19] Du X Pang TY . Is dysregulation of the hpa-axis a core pathophysiology mediating co-morbid depression in neurodegenerative diseases? Front Psychiatry. (2015) 6:32. doi: 10.3389/fpsyt.2015.00032, PMID: 25806005 PMC4353372

[20] Le JJ Yi T Qi L Li J Shao L Dong JC . Electroacupuncture regulate hypothalamic-pituitary-adrenal axis and enhance hippocampal serotonin system in a rat model of depression. Neurosci Lett. (2016) 615:66–71. doi: 10.1016/j.neulet.2016.01.004, PMID: 26773866

[21] Mosqueiro BP MP F Da RN . Increased levels of brain-derived neurotrophic factor are associated with high intrinsic religiosity among depressed inpatients. Front Psychiatry. (2019) 10:671. doi: 10.3389/fpsyt.2019.00671, PMID: 31572245 PMC6753839

[22] Yang J Pei Y YL P Jia J Shi C Yu Y . Enhanced antidepressant-like effects of electroacupuncture combined with citalopram in a rat model of depression. Evid Based Complement Alternat Med. (2013) 2013:107380. doi: 10.1155/2013/107380, PMID: 23737815 PMC3666268

[23] J ingguang X Xuejing W Jie W . Analysis of the efficacy of brain-computer interface-based electrical stimulation as an adjunctive therapy for geriatric depression. J Int Psychiatry. (2024) 51:808–10. doi: 10.13479/j.cnki.jip.2024.03.038

[24] Man SC BH H RM N XC Y Cheung H MP F . A pilot controlled trial of a combination of dense cranial electroacupuncture stimulation and body acupuncture for post-stroke depression. BMC Complement Altern Med. (2014) 14:255. doi: 10.1186/1472-6882-14-255, PMID: 25038733 PMC4223407

[25] Qingyan C Yi L Yue M Guo C Shanshan G Jiliang F . Comparison of brain effects of transcutaneous auricular vagus nerve stimulation therapy for treatment-resistant and non-treatment-resistant depression. Chin Imaging J Integrated Traditional Western Med. (2024) 22:4–11. doi: 10.3969j.issn.1672-0512.2024.01.002

[26] Gao Y Li T Lu Q Wang J Wang Y Wang L . Electroacupuncture for treating depression-related insomnia: a systematic review and meta-analysis. Front Psychiatry. (2025) 16:1610107. doi: 10.3389/fpsyt.2025.1610107, PMID: 40698050 PMC12279889

[27] Fang X Wang X Zheng W Han J Ge X . Efficacy and safety of electroacupuncture in patients with postpartum depression: a meta-analysis. Front Psychiatry. (2024) 15:1393531. doi: 10.3389/fpsyt.2024.1393531, PMID: 39056020 PMC11270539

[28] Ren C SR P Wang Z Kung S RB B Islam K . Transcranial electrical stimulation in treatment of depression: a systematic review and meta-analysis. JAMA Netw Open. (2025) 8:e2516459. doi: 10.1001/jamanetworkopen.2025.16459, PMID: 40531534 PMC12177679

[29] Salanti G . Indirect and mixed-treatment comparison, network, or multiple-treatments meta-analysis: many names, many benefits, many concerns for the next generation evidence synthesis tool. Res Synth Methods. (2012) 3:80–97. doi: 10.1002/jrsm.1037, PMID: 26062083

[30] Shamseer L Moher D Clarke M Ghersi D Liberati A Petticrew M . Preferred reporting items for systematic review and meta-analysis protocols (prisma-p) 2015: elaboration and explanation. BMJ. (2015) 350:g7647. doi: 10.1136/bmj.g7647, PMID: 25555855

[31] Hutton B Salanti G DM C Chaimani A CH S Cameron C . The prisma extension statement for reporting of systematic reviews incorporating network meta-analyses of health care interventions: checklist and explanations. Ann Intern Med. (2015) 162:777–84. doi: 10.7326/M14-2385, PMID: 26030634

[32] Eisma MC Janshen A Lenferink L . Content overlap analyses of icd-11 and dsm-5 prolonged grief disorder and prior criteria-sets. Eur J Psychotraumatol. (2022) 13:2011691. doi: 10.1080/20008198.2021.2011691, PMID: 35096286 PMC8794064

[33] Association CM House CMJP Practice CSOG Psychiatry DDCG Association EBOC Disease EGOG . Guideline for primary care of major depressive disorder (2021). Chin J Gen Practitioners. (2021) 20:1249–60.

[34] Furukawa TA Akechi T Azuma H Okuyama T Higuchi T . Evidence-based guidelines for interpretation of the hamilton rating scale for depression. J Clin Psychopharmacol. (2007) 27:531–4. doi: 10.1097/JCP.0b013e31814f30b1, PMID: 17873700

[35] Sterne J Savovic J MJ P RG E NS B Boutron I . Rob 2: a revised tool for assessing risk of bias in randomised trials. BMJ. (2019) 366:l4898. doi: 10.1136/bmj.l4898, PMID: 31462531

[36] Higgins JP SG T JJ D Altman DG . Measuring inconsistency in meta-analyses. BMJ. (2003) 327:557–60. doi: 10.1136/bmj.327.7414.557, PMID: 12958120 PMC192859

[37] Chaimani A JP H Mavridis D Spyridonos P Salanti G . Graphical tools for network meta-analysis in stata. PloS One. (2013) 8:e76654. doi: 10.1371/journal.pone.0076654, PMID: 24098547 PMC3789683

[38] Egger M SG D Schneider M Minder C . Bias in meta-analysis detected by a simple, graphical test. BMJ. (1997) 315:629–34. doi: 10.1136/bmj.315.7109.629, PMID: 9310563 PMC2127453

[39] Puhan MA HJ S MH M Li T Brignardello-Petersen R JA S . A grade working group approach for rating the quality of treatment effect estimates from network meta-analysis. BMJ. (2014) 349:g5630. doi: 10.1136/bmj.g5630, PMID: 25252733

[40] Mccarron RM Shapiro B Rawles J Luo J . epression. Ann Intern Med. (2021), 174:ITC65–ITC80. doi: 10.7326/AITC202105180, PMID: 33971098

[41] Klonsky ED AM M Saffer BY . Suicide, suicide attempts, and suicidal ideation. Annu Rev Clin Psychol. (2016) 12:307–30. doi: 10.1146/annurev-clinpsy-021815-093204, PMID: 26772209

