# Supplementary material for: Effects of different electrical stimulation on depression: a protocol for systematic review and network meta-analysis
Source: Front Psychiatry. 2025 Nov 21;16:1684994. doi: 10.3389/fpsyt.2025.1684994 (PMC12679154; doi:10.3389/fpsyt.2025.1684994)
Supplement: Supplementary file 2 [file DataSheet1.docx]

**Table 1. Search strategy for the English databases.**

| **Order** | **strategy** |
| --- | --- |
| #1 | (Depressive Disorders[MeSH Terms]) |
| #2 | (Depression[Title/Abstract]) OR (Disorder, Depressive[Title/Abstract]) OR (Disorders, Depressive[Title/Abstract]) OR (Neurosis, Depressive[Title/Abstract]) OR (Depressive Neuroses[Title/Abstract]) OR (Depressive Neurosis[Title/Abstract]) OR (Neuroses, Depressive[Title/Abstract]) OR (Depression, Endogenous[Title/Abstract]) OR (Depressions, Endogenous[Title/Abstract]) OR (Endogenous Depression[Title/Abstract]) OR (Endogenous Depressions[Title/Abstract]) OR (Melancholia[Title/Abstract]) OR (Melancholias[Title/Abstract]) OR (Unipolar Depression[Title/Abstract]) OR (Depression, Unipolar[Title/Abstract]) OR (Depressions, Unipolar[Title/Abstract]) OR (Unipolar Depressions[Title/Abstract]) OR (Depressive Syndrome[Title/Abstract]) OR (Depressive Syndromes[Title/Abstract]) OR (Syndrome, Depressive[Title/Abstract]) OR (Syndromes, Depressive) OR (Depression, Neurotic[Title/Abstract]) OR (Depressions, Neurotic[Title/Abstract]) OR (Neurotic Depression[Title/Abstract]) OR (Neurotic Depressions[Title/Abstract]) |
| #3 | #1 OR #2 |
| #4 | (Electric Stimulation[MeSH Terms]) |
| #5 | (Electrical Stimulation[Title/Abstract]) OR (Electrotherapy[Title/Abstract]) OR (neuromuscular electrical stimulation[Title/Abstract]) OR (NMES[Title/Abstract]) OR (functional electrical stimulation[Title/Abstract]) OR (FES[Title/Abstract]) OR (Stimulation electrode[Title/Abstract]) OR (Transcutaneous electrical stimulation[Title/Abstract]) OR (Transcutaneous electric stimulation[Title/Abstract]) OR (Transcutaneous electrical nerve stimulation[Title/Abstract]) OR (TENS[Title/Abstract]) OR (Percutaneous electric nerve stimulation[Title/Abstract]) OR (Transcutaneous electrical acupoint stimulation[Title/Abstract]) OR (TEAS[Title/Abstract]) OR (Electrical muscle stimulation[Title/Abstract]) OR (Electric muscle stimulation[Title/Abstract]) OR (Transdermal electrostimulation[Title/Abstract]) OR (Electroacupuncture[Title/Abstract]) OR (Electrical acupuncture[Title/Abstract]) OR (Electric acupuncture[Title/Abstract]) OR (EA[Title/Abstract]) |
| #6 | #4 OR #5 |
| #7 | (((((Randomized Controlled Trial[Title/Abstract]) OR (Controlled Clinical Trial[Title/Abstract])) OR (Random[Title/Abstract])) OR (Randomization[Title/Abstract])) OR (Random Allocation[Title/Abstract])) OR (Random[Title/Abstract]) |
| #8 | #3 AND #6 AND #7 |

**Table 2. Search strategy for the Chinese databases.**

| **Order** | **strategy** |
| --- | --- |
| #1 | '电刺激'+'电疗'+'神经电刺激'+'肌肉电刺激'+'神经肌肉电刺激'+'功能性电刺激'+'刺激电极'+'经皮电刺激'+'经皮穴位电刺激'+'经皮神经电刺激'+'电针'+'中频电疗仪' |
| #2 | '抑郁症'+'郁病'+'郁证' |
| #3 | 随机对照试验"OR"随机对照研究"OR"随机对照"OR"RCT"OR"随机" |
| #4 | #1 AND #2 AND #3 |
